# A New Double Stranded RNA Suppresses Bladder Cancer Development by Upregulating p21^**Waf1/CIP1**^ Expression

**DOI:** 10.1155/2015/304753

**Published:** 2015-03-30

**Authors:** Chenghe Wang, Qiangqiang Ge, Zhong Chen, Jia Hu, Fan Li, Xiaodong Song, Hua Xu, Zhangqun Ye

**Affiliations:** ^1^Department of Urology, Tongji Hospital, Tongji Medical College, Huazhong University of Science and Technology, No. 1095 Jie Fang Avenue, Wuhan, Hubei 430030, China; ^2^Institute of Urology of Hubei Province, No. 1095 Jie Fang Avenue, Wuhan, Hubei 430030, China

## Abstract

We have previously demonstrated that miR-1180-5p has potent ability to upregulate p21 expression by targeting promoter and inhibit bladder cancer. This prompted us to conjecture that a candidate dsRNA (dsP21-397) with perfect complementarity to the miR-1180-5p target site of p21 promoter may also trigger p21 expression. Transfection of dsP21-397 into T24 and EJ cells significantly activated p21 expression at 72 h and the activation presented in a time-course and dose-dependent manner. Moreover, the p21-activated activities of dsP21-397 and miR-1180-5p are not significantly different. Overexpression of p21 downregulated Cyclin D1, CDK4/6, and Cyclin A2 expression, and thereby induced cell cycle arrest and inhibited proliferation. Moreover, dsP21-397 suppressed bladder cancer largely depended on manipulating p21. In conclusion, our study identifies a pair of miRNA-dsRNA mediating endogenous p21 overexpression.

## 1. Introduction

Bladder cancer (BC) represents one of the most common tumors diagnosed in the United States, with an estimated annual incidence of 74,690 new cases and 15,580 deaths in 2014 [1]. The main characteristic of cancer cells is their increased proliferative activity, which is often caused by aberrations in cell cycle regulation [2]. The p21^WAF1/CIP1^ (p21) is a broad-acting cyclin-dependent kinase (CDK) inhibitor that binds and inhibits Cyclin-CDK complexes and thereby mediates cell cycle in the G1 phase and leads to proliferation arrest [3, 4]. Therefore, upregulation of p21 is an ideal method of gain-of-function manipulation to suppress cancer cell growth.

RNA interference (RNAi) is an evolutionary conserved gene silencing mechanism in which small RNAs, such as endogenous microRNAs (miRNAs) or exogenous double stranded RNAs (dsRNAs), target homologous mRNA sequences to degrade the mRNAs or inhibit their translation [5, 6]. In contrast, increasing evidences have emerged that miRNAs and dsRNAs can also function to upregulate specific genes expression by targeting promoter, a phenomenon termed as RNA activation (RNAa) [7, 8]. As tumorigenesis may result from functional silence of tumor suppressor genes, enforced expression of these genes by RNAa would otherwise offer great potential for cancer therapy.

Place et al. reported that E-cadherin can be induced through RNAa after transfection of mature miR-373 or the corresponding dsRNA (dsEcad-640) which is fully complementary to promoter sequences [7]. We have also shown that miR-1180-5p holds the capacity to upregulate p21 expression and inhibit BC cells [9]. However, whether a homologous dsRNA perfectly complementary to putative miR-1180-5p target site of p21 promoter can activate p21 expression and suppress BC remains unexplored.

In the present study, we transfect a candidate dsRNA into BC cells and examine the p21 expression. Our results show that the dsRNAs with complete complementarity to the same p21 promoter sequences also significantly inhibit BC cells proliferation and clonogenicity.

## 2. Materials and Methods

### 2.1. dsRNAs

All the RNA duplexes which possessed 2-nucleotide 3′ overhangs were chemically synthesized by GenePharma (Shanghai, China). A small interfering RNA (siP21) was used to silence p21 expression and a dsRNA lacking significant homology to all known human sequences (dsControl) was used as a nonspecific control [7, 10]. All the custom dsRNA sequences are listed in Supplementary Table S1 (available online at http://dx.doi.org/10.1155/2015/304753).

### 2.2. Cell Culture and Transfection

Human BC cell lines T24 and EJ (ATCC) were cultured in RPMI 1640 medium (Hyclone, USA) supplemented with 10% fetal bovine serum (Gibco, USA) in a humidified atmosphere with 5% CO_2_ at 37°C. The day before transfection, cells were plated to about 60% confluency in medium without antibiotics. dsRNA was transfected at indicated concentrations by using Lipofectamine RNAiMax (Invitrogen, USA) according to the manufacturer's instructions. Medium was replaced 8 hours later after transfection and then changed daily.

### 2.3. RNA Isolation and Real-Time PCR

Total cellular RNA from BC cells was extracted by using Trizol reagent (Invitrogen, USA). 500 ng of RNA was used for cDNA synthesis according to the protocol provided by Takara reverse transcription kit (Takara, China). The resulting cDNA was amplified by SYBR Premix Ex Taq II (Takara, China) conducted on the Mx3000P system (Stratagene, USA). The primers included in this study were provided by Invitrogen (Shanghai, China) and listed in Supplementary Table S2. GAPDH was used as internal control to determine the relative expression of target genes' mRNA. All reactions were performed in triplicates.

### 2.4. Protein Extraction and Western Blot Analysis

Total proteins were extracted using RIPA lysis buffer supplemented with protease inhibitor Cocktail (Roche, Switzerland). Protein concentrations were calculated by using BCA protein assay kit (Beyotime, China). Equivalent amounts of protein samples (50 *μ*g) of each group were separated by 10% sodium dodecyl sulfate polyacrylamide gel electrophoresis (SDS-PAGE) and then transferred to a PVDF membrane. Membranes were blocked in 5% nonfat dried milk and incubated with the primary antibodies overnight at 4°C. After several washes, membranes were incubated for 1 h at room temperature with corresponding second antibody and detected by enhanced chemiluminescence (ECL) assay kit (Millipore, USA). The primary antibodies were as follows: anti-p21 (1/2000) (Cell Signaling Technology, USA), anti-Cyclin D1 (1/2000) (Affinity, USA), anti-CDK4 (1/1000) (Affinity, USA), anti-CDK6 (1/2000) (Affinity, USA), anti-Cyclin A2 (1/400) (Boster, China), anti-GAPDH (1/500) (Boster, China), and anti-*α*-tublin (1/500) (Boster, China).

### 2.5. Clonogenic Survival Assay

After transfection of indicated dsRNA for 24 h, cells were trypsinized, counted, and reseeded in six-well plates at the density of 1000 cells/well for 10 days with complete medium. The medium was changed every 3 days to maintain the cells growth. The colonies were fixed and stained with 0.5% crystal violet (Sigma, USA) for 30 min at room temperature. The colony formation rate was calculated using the following equation: colony formation rate = number of colonies/number of seeded cells × 100%.

### 2.6. Cell Cycle Analysis

72 h after transfection, cells were harvested by trypsinization and fixed with 70% ethanol at 4°C overnight. Then the cells were washed and incubated with RNase A (0.1 mg/mL) for 30 min at 37°C. Cellular DNA was stained with propidium iodide (PI) (0.05 mg/mL) and analyzed on a FACSort flow cytometer (BD Biosciences, USA). All experiments were performed in triplicate and a total of 10,000 events were analyzed for each sample. The data were processed by CELL Quest software (BD Biosciences, USA).

### 2.7. Cell Proliferation Assay

Cell proliferation was determined using the CellTiter 96 AQ_ueous_ One Solution Cell Proliferation Assay kit (Promega, USA) according to the manufacturer's protocol. Briefly, cells were transfected with dsRNA in a 6-well plate. The following day, the cells were trypsinized and seeded at 1000 cells/well into a new 96-well plate. The plates were then incubated for 5 days and cell growth was measured at daily intervals from the next day to the fifth day. At each time point, 20 *μ*L of CellTiter 96 AQ_ueous_ One Solution was added to each well and incubated for 2 hour at 37°C. Absorbance was measured on a microplate reader (Bio-Rad, USA) at 490 nm.

### 2.8. Statistical Analysis

All data were expressed as the mean ± standard deviation (SD) for three independent experiments. Differences between groups were analyzed by *t*-tests using SPSS version 13.0 software (SPSS Inc., Chicago, IL, USA). *P* value < 0.05 was considered to be statistically significant.

## 3. Results

### 3.1. dsP21-397 Targets p21 Promoter to Induce Its Expression

**Figure 1:**
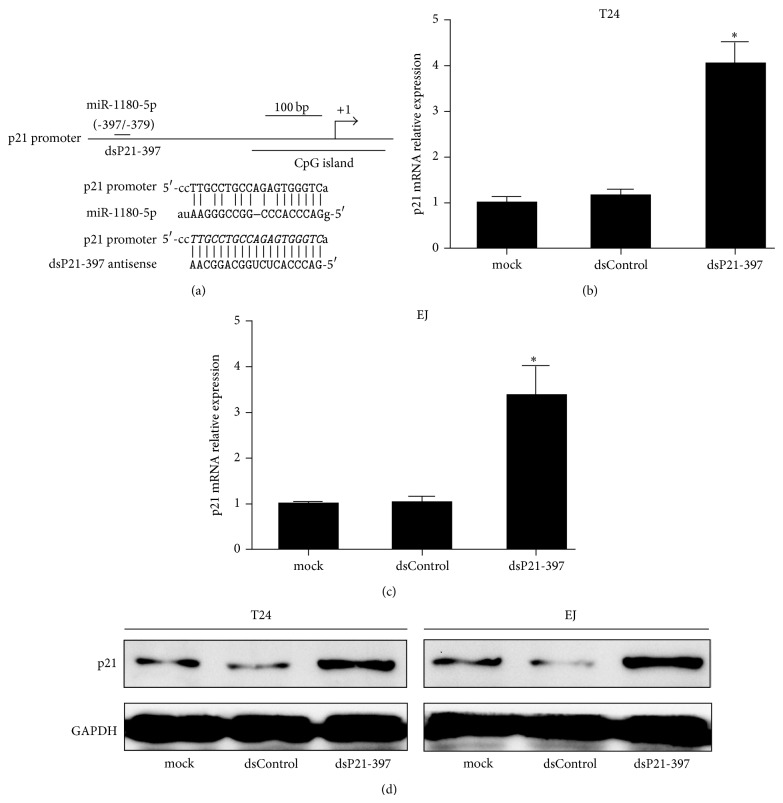
dsP21-397 induces p21 expression by targeting a complementary promoter sequence in BC cells. (a) Schematic representation of the p21 promoter and dsP21-397 target site. Sequence of the miR-1180-5p target site located at nucleotide -397 relative to the transcription start site. (b and c) Expression of p21 and GAPDH mRNA levels was assessed by real-time PCR. GAPDH served as a loading control. T24 and EJ cells were transfected with 50 nM of the indicated dsRNAs for 72 h. mock sample was transfected in the absence of dsRNA. ^∗^ *P* < 0.001 compared to mock and dsControl groups. (d) Induction of p21 protein expression was detected by Western blot analysis. GAPDH levels were also detected and served as a loading control.

We have reported p21 is susceptible to gene induction with miR-1180-5p by targeting its promoter sequences at -397/-379 relative to transcription starting site (Figure 1(a)) [9]. It is speculated that a candidate dsRNA (dsP21-397) fully complementary to the putative miR-1180-5p target site may also have the ability to activate p21 expression (Figure 1(a)). Hereby we transfected synthetic dsP21-397 into T24 and EJ cells and analyzed p21 expression 72 h later. Compared to mock transfection, dsP21-397 caused a 4.03- and 3.35-fold induction in p21 mRNA expression, respectively (Figures 1(b) and 1(c)). This induction was further confirmed by western blot assessment (Figure 1(d)). These results suggest that dsP21-397 holds the capacity to induce p21 expression by targeting promoter.

### 3.2. Properties of dsP21-397 Mediated p21 Gene Expression

**Figure 2:**
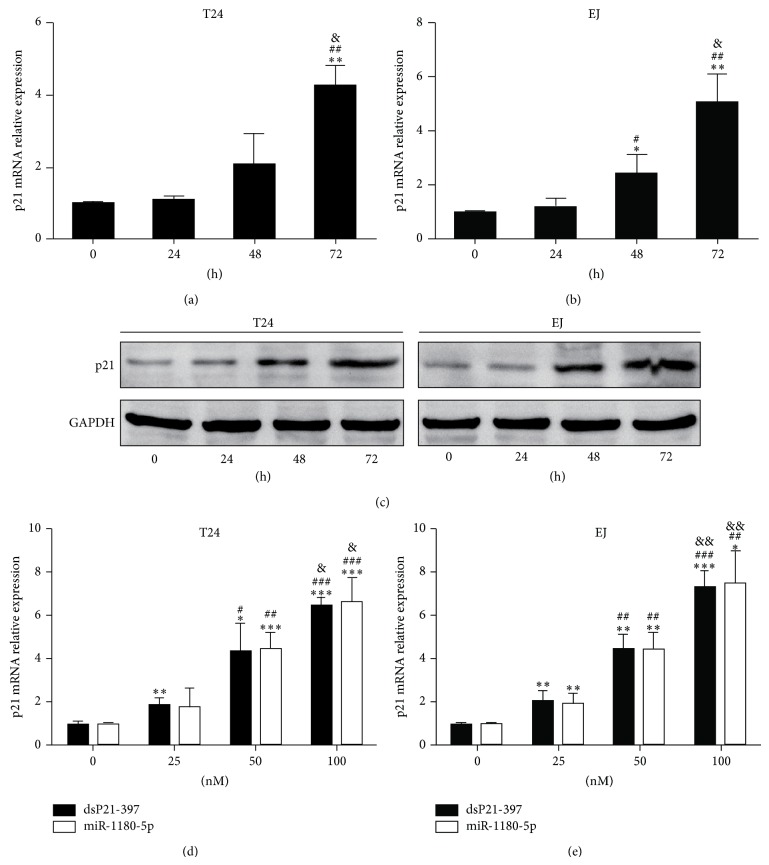
Characterization of dsP21-397-mediated upregulation of p21 expression. (a) and (b) Time-course of dsP21-397-mediated upregulation of p21 expression. T24 and EJ cells were transfected with dsP21-397 at 50 nM. Cells were subjected to real-time PCR at the indicated time points. ^∗^ *P* < 0.01 and ^∗∗^ *P* < 0.001 compared to 0 h; ^#^ *P* < 0.05 and ^##^ *P* < 0.001 compared to 24 h; ^&^ *P* < 0.01 compared to 48 h. (c) Expression of p21 protein was assessed by western blotting analysis. GAPDH served as a loading control. Cells were transfected with 50 nM dsP21-397 for the indicated periods of time. (d) and (e) Dose-dependent upregulation of p21 mRNA following transfection with dsP21-397 or miR-1180-5p at the indicated concentrations. Cells were subjected to real-time PCR at 72 h. GAPDH served as a loading control. ^∗^ *P* < 0.05, ^∗^ *P* < 0.01, and ^∗∗∗^ *P* < 0.001 compared to corresponding 0 nM; ^#^ *P* < 0.05, ^##^ *P* < 0.01, and ^###^ *P* < 0.001 compared to corresponding 25 nM; ^&^ *P* < 0.05 and ^&&^ *P* < 0.01 compared to corresponding 50 nM.

Next, we evaluated the time-course effects of dsP21-397-mediated upregulation of p21 in BC cell lines. As shown in Figures 2(a) and 2(b), induction of p21 mRNA expression in both T24 and EJ cells began to emerge 48 h after transfection and the level continued to increase at 72 h. This time-course feature was further confirmed by immunoblot analysis (Figure 2(c)). The results suggest that enhanced p21 expression may be owing to the direct effect of RNAa. Moreover, to compare the activities of miR-1180-5p and dsP21-397 on activating p21, expression of the gene's mRNA was quantified by the dose-dependent experiments at 72 h following the two types of small RNAs transfection. Apparently, higher concentrations of miR-1180-5p or dsP21-397 caused more p21 mRNA expression, but the mRNA levels were not significantly different at the same concentrations of the two activators (Figures 2(d)-2(e) and Figure S1).

### 3.3. dsP21-397 Induces BC Cell Cycle Arrest, Inhibits Proliferation, and Suppresses Colony Formation

**Figure 3:**
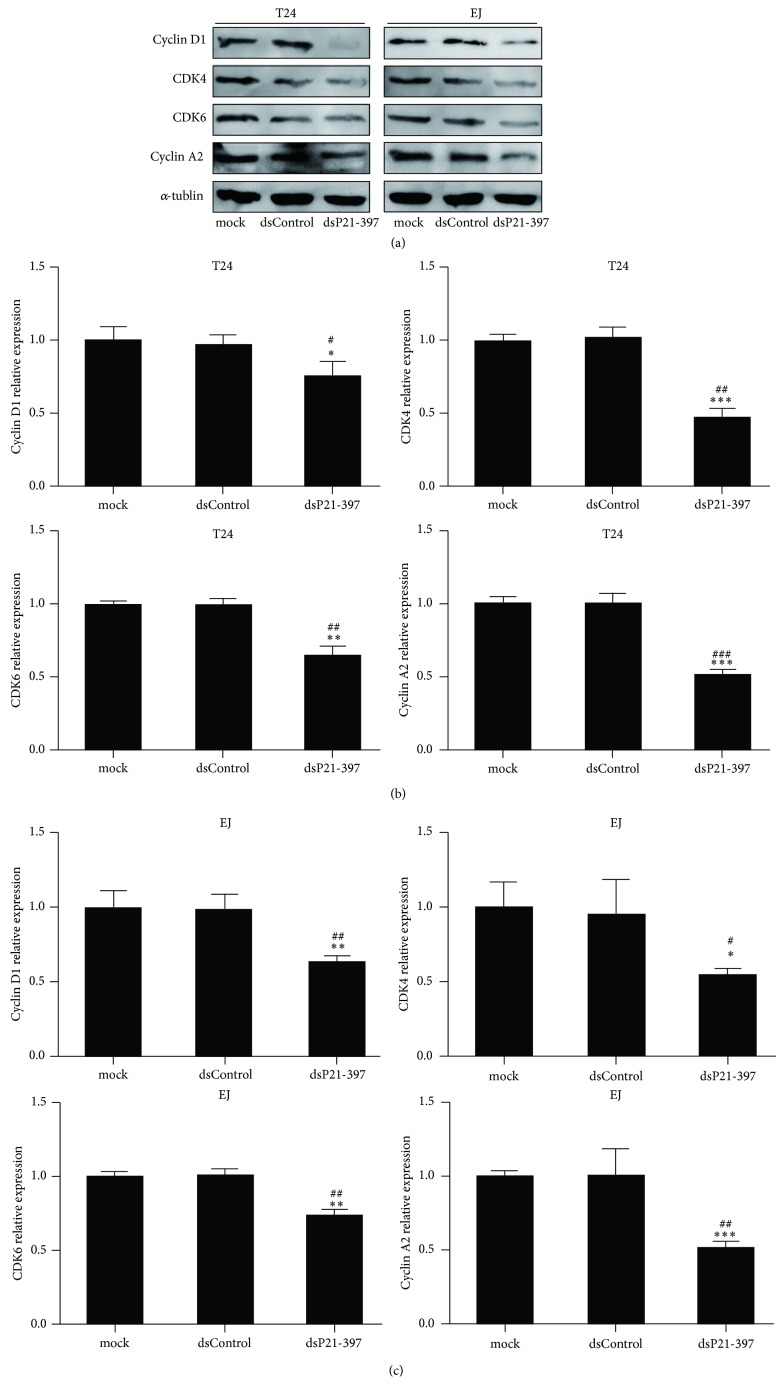
dsP21-397 inhibits cyclins and CDKs expression in BC cell lines. T24 and EJ cells were transfected with 50 nM of the indicated dsRNAs for 72 h. mock sample was transfected in the absence of dsRNA. (a) Expression of Cyclin D1, CDK4/6 and Cyclin A2 protein in BC cells was detected by Western blot. *α*-tublin served as a loading control. (b) and (c) Densitometric analysis quantification of Cyclin D1, CDK4/6, and Cyclin A2 proteins expression in T24 and EJ cells. ^∗^ *P* < 0.05, ^∗∗^ *P* < 0.01, and ^∗∗∗^ *P* < 0.001 compared to mock group; ^#^ *P* < 0.05, ^##^ *P* < 0.01, and ^###^ *P* < 0.001 compared to dsControl group.

To investigate the effect of p21 upregulation induced by dsP21-397 on BC cells development, we analyzed the expression of Cyclin-CDK which manipulates the cell cycle progression. As shown in Figure 3(a), compared with mock and dsControl groups, western blot assay indicated that Cyclin D1, CDK4, CDK6, and Cyclin A2 were significantly decreased at 72 h after dsP21-397 transfection in T24 and EJ cells. The decreases were further verified by densitometric analysis of the immunoblots (Figures 3(b) and 3(c)).

**Figure 4:**
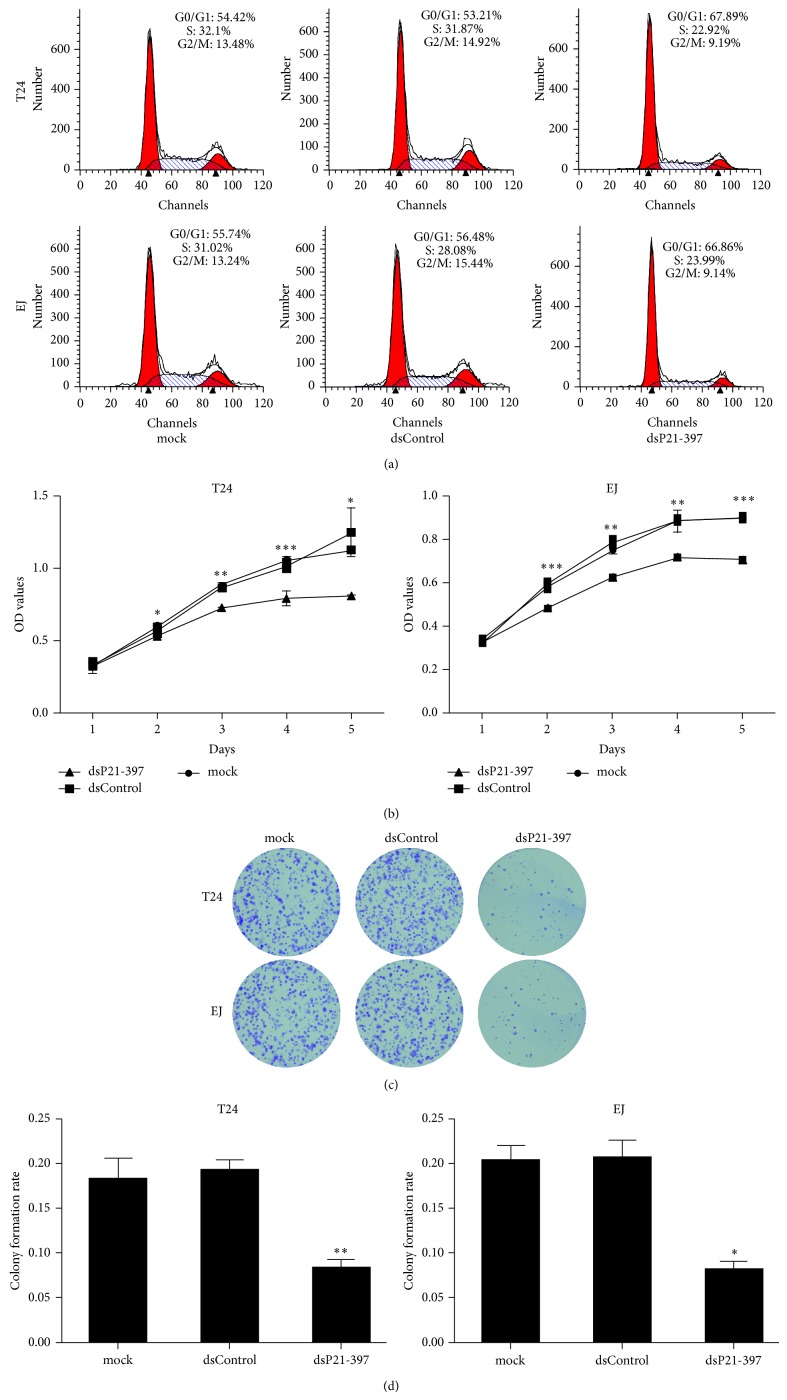
dsP21-397 induces BC cells cycle arrest and inhibits proliferation. T24 and EJ cells were transfected with 50 nM of the indicated dsRNAs for 72 h. mock sample was transfected in the absence of dsRNA. (a) Cell cycle distribution was conducted using flow cytometry with PI stained. (b) Viable cells were measured from day 1 to 5 following transfection using the CellTiter 96 AQ_ueous_ One Solution Cell Proliferation Assay kit. Results were plotted as OD values. ^∗^ *P* < 0.05, ^∗∗^ *P* < 0.01, and ^∗∗∗^ *P* < 0.001 compared to mock and dsControl groups at the same time point. (c) Representative photographs of colony formation assay. (d) Quantification of the cell colonies formation. ^∗^ *P* < 0.01 and ^∗∗^ *P* < 0.001 compared to mock and dsControl groups.

Then we performed flow cytometry to measure cell cycle distribution. In both tested cell lines, transfection with dsP21-397 resulted in a significant increase in the G0/G1 population and a corresponding decrease in the S phase population compared with mock and dsControl treatments (Figure 4(a)). Besides, CellTiter 96 AQ_ueous_ One Solution Cell Proliferation Assay demonstrated that compared to mock and dsControl groups, both T24 and EJ cells transfected dsP21-397 exhibited progressive retarded growth from day 2 following transfection (Figure 4(b)). However, mock transfected cells maintained similar growth with cells transfected with control dsRNA (Figure 4(b)). We further checked whether transfection of dsP21-397 exerts an inhibitory influence on cell proliferation ability in BC cells. Apparently, colony formation assay revealed that dsP21-397 transfected cells formed colonies significantly fewer in number and smaller in size (Figure 4(c)). Moreover, the colony rates of dsP21-397 transfected cells were remarkably lower than mock and dsControl treatments (Figure 4(d)). These results are in agreement with previous studies in which activation of p21 leads to G0/G1 arrest and decline in cell proliferation [11, 12].

### 3.4. dsP21-397 Decreases Cyclin D1-CDK4/6 and Cyclin A2 Expression by Activating p21

**Figure 5:**
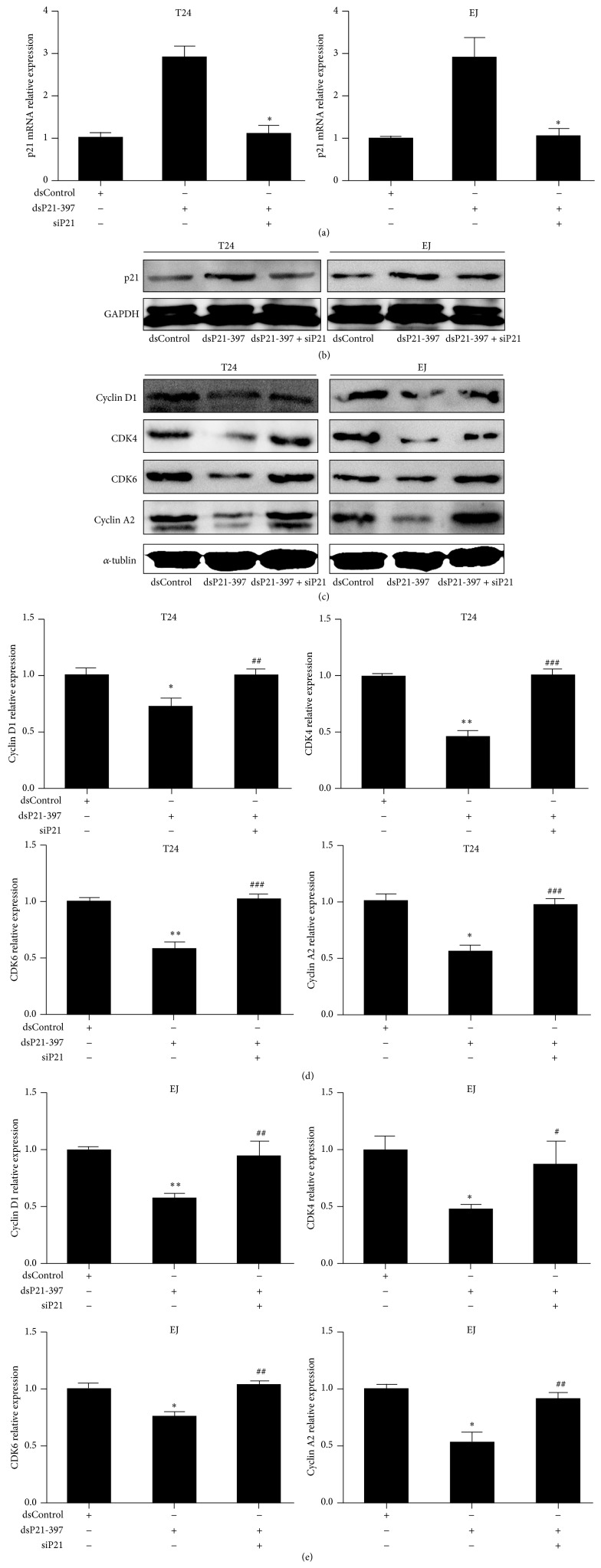
dsP21-397 inhibits cyclins and CDKs expression mainly by regulating p21. Cells were transfected with 50 nM of the indicated dsRNAs for 72 h. (a) Expression of p21 was assessed by real-time PCR. GAPDH served as a loading control. ^∗^ *P* < 0.001 compared to dsP21-397 group. (b) Induction of p21 protein expression was detected by Western blot analysis. GAPDH were also detected and served as a loading control. (c) Expression of Cyclin D1, CDK4/6, and Cyclin A2 protein was detected by Western blot. *α*-tublin served as a loading control. (d) and (e) Densitometric analysis quantification of Cyclin D1, CDK4/6, and Cyclin A2 proteins expression in T24 and EJ cells. ^∗^ *P* < 0.01 and ^∗∗^ *P* < 0.001 compared to dsControl group; ^#^ *P* < 0.05, ^##^ *P* < 0.01, and ^###^ *P* < 0.001 compared to dsP21-397 group.

To validate that dsP21-397 suppresses Cyclin-CDK expression mainly by upregulating p21, we silenced p21 expression by transfecting small interfering RNA siP21 in T24 and EJ cells [13]. The results showed that cotransfection of dsP21-397 and siP21 in BC cells markedly abrogated the activating effect mediated by dsP21-397 at both mRNA and protein levels at 72 h (Figures 5(a) and 5(b)). Then, we examined the expression of p21 downstream effectors in BC cells. As seen from Figure 5(c), dsP21-397 failed to downregulate Cyclin D1, CDk4/6, and Cyclin A2 expression after siP21 cotreatments. Densitometric analysis quantification of immunoblots further proved that (Figures 5(d) and 5(e)). This suggests that dsP21-397 inhibits Cyclin D1, CDK4/6, and Cyclin A2 expression largely depending on modulating p21.

### 3.5. dsP21-397 Inhibits BC Cells Mainly by Upregulation of p21

**Figure 6:**
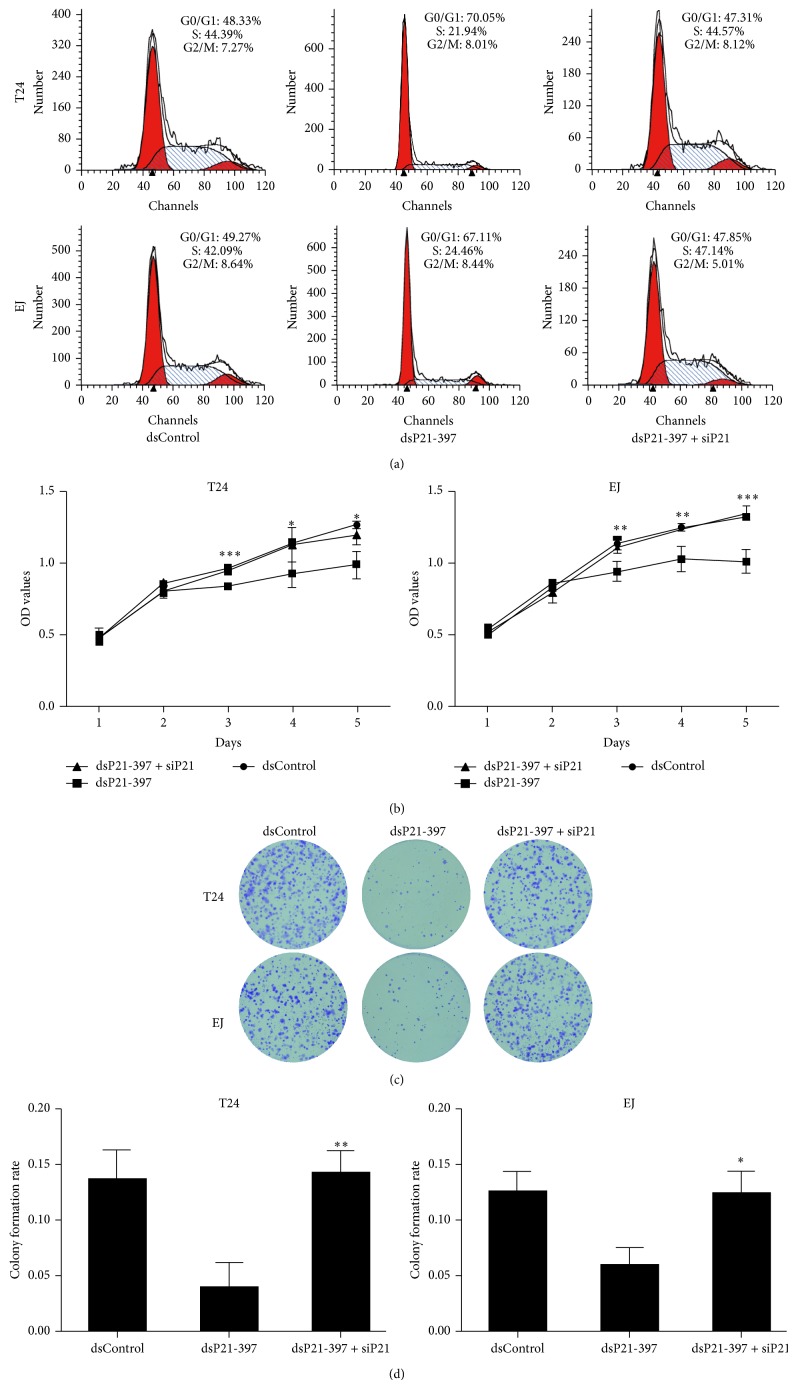
dsP21-397 suppresses BC cells largely depending on manipulating p21 expression. (a) Cell cycle distribution was conducted using flow cytometry with PI stained. (b) Viable cells were measured from day 1 to 5 following transfection using the CellTiter 96 AQ_ueous_ One Solution Cell Proliferation Assay kit. Results were plotted as OD values. ^∗^ *P* < 0.05, ^∗∗^ *P* < 0.01, and ^∗∗∗^ *P* < 0.001 compared to dsP21-397 group at the same time point. (c) Representative photographs of colony formation assay. (d) Quantification of the cell colonies formation. ^∗^ *P* < 0.05 and ^∗∗^ *P* < 0.01 compared to dsP21-397 group.

Next, we verify whether depletion of p21 could affect the inhibitory function of dsP21-397 in T24 and EJ cells. Remarkably, p21 silencing significantly attenuated G0/G1 cycle arrest mediated by dsP21-397 in both cell lines (Figure 6(a)). Moreover, the antiproliferative effect resulting from Cyclin D1, CDK4/6, and Cyclin A2 downregulation was evidently alleviated by p21 silencing (Figure 6(b)). In addition, the colony formation ability of the BC cells was also restored (Figures 6(c) and 6(d)). Taken together, these experiments strongly implied that p21 is mainly responsible for BC cells suppression by dsP21-397.

## 4. Discussion

In the present study, we identify that a synthetic dsRNA (dsP21-397), which is completely complementary to putative miR-1180-5p target site of p21 promoter, exhibits considerable potency to activate p21 expression in BC cells. And this activation presents a time-course and dose-dependent effect. Moreover, dsP21-397 possesses a similar capacity with miR-1180-5p to enhance p21 expression. Several Cyclin-CDK genes (Cyclin D1, CDK4/6, and Cyclin A2) associated with cell cycle are downregulated following dsP21-397 transfection. Hereby, transfection of dsP21-397 causes BC cells' cycle arrest and impedes proliferation. In addition, this dsRNA's antitumor function is mainly achieved by regulating p21 expression.

One distinctive feature of cancer cells is their sustained proliferative activity; thus blockade of cell cycle is regarded as effective strategy for cancer therapy [14]. Cell cycle is controlled by Cyclin-CDK. Inhibition of Cyclin D1-CDK4/6 complexes activity elicits reduction in G0/G1-to-S phase progression [15]. Furthermore, Cyclin A2 plays a decisive role in regulation of S phase and its deficiency will make cells accumulation in G0/G1 and decreases entry into S phase [16]. It is now well known that p21 is a powerful CDK inhibitor involved in many antigrowth pathways including promoting cell cycle arrest [17]. The p21 protein has a cyclin binding motif and inhibits Cyclin-CDK complexes, such as Cyclin D1-CDK4/6 and Cyclin A2-CDK2, by direct interaction [18]. By the way, we have shown that several miRNAs mediated p21 overexpression potently accelerates BC cells apoptosis [9]. However, saRNAs also own the proapoptotic effect on BC cells due to the similar approach [19–21].

Earlier studies indicated that p21 is frequently low expressed in BC cells and correlates with prognosis [22, 23]; enhancement of its expression by RNAa would undoubtedly exert potent antitumor capacity [9, 19, 24]. Moreover, we and others have reported that p21 expression could be significantly induced by multiple dsRNA or miRNAs in variable levels by targeting different regions of promoter [9, 25].

RNAi is a posttranscriptional gene silencing mechanism while RNAa functions at the transcriptional level [8, 26]. RNAi and RNAa regulate target genes either negatively or positively, presenting opposite effects. Unlike RNAi, the exact mechanism of RNAa is not well-characterized. However, to our knowledge, this RNAa phenomenon required both transcriptional and epigenetic alterations. The dsRNA is first loaded onto a cytoplasmic Argonaute. Then the sense strand is removed and an active Argonaute-RNA complex formed. The complex is further proceeded to bind complementary promoter DNA guided by the antisense strand. Afterwards, histone modifying enzymes are enriched at the promoter and activate transcription via epigenetic modifications [27, 28].

As we know, high complementarity of sequences between miRNA/dsRNA and its target promoter is essential for RNAa [29]. Previous studies have reported that endogenous miRNAs partially complementary to promoters still possess the ability to activate genes but excess mismatch is not tolerable [7, 9, 30]. So it can be concluded that a synthetic dsP21-397 fully complementary to p21 promoter would induce p21 expression more easily. Surprisingly, even with a higher complementarity to p21 promoter, dsP21-397 did not induce more p21 expression than miR-1180-5p at the same transfecting concentration in our study. The exact mechanism is unclear and future research is needed.

Recently, noncoding RNAs, such as enhancer RNAs (eRNAs) and natural antisense transcripts (NATs), have been recognized to regulate genes expression by interacting with promoter. However, their gene modulating modes are distinct from RNAa. Generally, eRNAs are transcribed from enhancers and contribute to enhancers function by mediating enhancer-promoter interaction [31, 32]. Sequences of NATs are complementary to that of sense strand which is defined as mRNA and encode proteins [33]. Besides, NATs can be found in promoters, introns, exons, and untranslated regions of the genome [34]. Functionally, NATs can suppress transcription by chromatin remodeling, transcriptional interference, RNA masking, and so on [34].

BC arising contains numerous genetic alternations, such as inactivation of tumor suppressor genes [35, 36]. Therefore, reactivation of specific tumor suppressor p21 will surely offer a promising method for cancer therapeutics. Additionally, Li et al. have demonstrated that RNAa is safe for manipulating certain gene expression without altering expression profile of other nonspecific genes [8].

## 5. Conclusions

Taken together, our results provide evidences that dsP21-397 stimulated p21 expression by targeting promoter-derived sequences which is identical to miR-1180-5p target site. Moreover, dsP21-397 also has the capacity to induce cell cycle arrest and inhibits proliferation of BC cells. Our study first identifies a pair of miRNA-dsRNA which both mediate endogenous p21 overexpression and suppress BC.

## Supplementary Material

Supplementary Table S1: All dsRNAs used in present study werechemically synthesized with dTdT 3'overhangs. The dsP21-397 complementary to the p21 promoter at sequences position -397 relative to the transcription starting site was used to activate p21 expression. The control dsRNA (dsControl) was designed to lack significant homology to all known human genes' sequences. The dsRNA (siP21) was used to silence p21 expression by targeting p21 mRNA 3' untranslated regions.
Supplementary Table S2: The primes were used to detect p21 and GAPDH mRNA relative expression levels in BC cell lines by real-time PCR.
Figure S1: In order to compare the activating activities between miR-1180-5p and dsP21-397, we transfected T24 and EJ cells with miR-1180-5p or dsP21-397 at 0nM, 25nM, 50nM and 100nM, respectively. 72 h later, p21 mRNA was determined by real-time PCR and the miR-1180-5p induced p21 mRNA expression levels were normalized to dsP21-397 groups at the same concentrations. As shown in Figure S1A and S1B, the p21 expression was not significantly different between miR-1180-5p and dsP21-397 at the same transfecting concentrations in T24 and EJ cells.
