# Supplementary material for: A New Double Stranded RNA Suppresses Bladder Cancer Development by Upregulating p21Waf1/CIP1 Expression
Source: Biomed Res Int. 2015 Mar 30;2015:304753. doi: 10.1155/2015/304753 (PMC4396018; doi:10.1155/2015/304753)

Supplementary Tables

Supplementary Table S1. Sequences for double-stranded RNAs used in present study.

	Sequences (5'-3')
dsP21-397 Sense	UUGCCUGCCAGAGUGGGUC[dT][dT]
dsP21-397 Antisense	GACCCACUCUGGCAGGCAA[dT][dT]
dsControl Sense	ACUACUGAGUGACAGUAGA[dT][dT]
dsControl Antisense	UCUACUGUCACUCAGUAGU[dT][dT]
siP21 Sense	CUUCGACUUUGUCACCGAG[dT][dT]
siP21 Antisense	CUCGGUGACAAAGUCGAAG[dT][dT]

Supplementary Table S2. Primers used for real-time PCR.

	Sequences (5’-3’)
p21 (forward)	GCCCAGTGGACAGCGAGCAG
p21 (reverse)	GCCGGCGTTTGGAGTGGTAGA
GAPDH (forward)	TCCCATCACCATCTTCCA
GAPDH (reverse)	CATCACGCCACAGTTTCC

GAPDH = glyceraldehide-3-phosphate dehydrogenase.

Supplementary Figure legends

Figure S1. The p21-activated activities of dsP21-397 or miR-1180-5p in BC cells. Cells were transfected with dsP21-397 or miR-1180-5p at the indicated concentrations. (**A**) and (**B**) Expression of p21 mRNA was evaluated by real-time PCR and normalized to dsP21-397 group at the indicated concentrations. GAPDH served as a loading control.

Figure S1

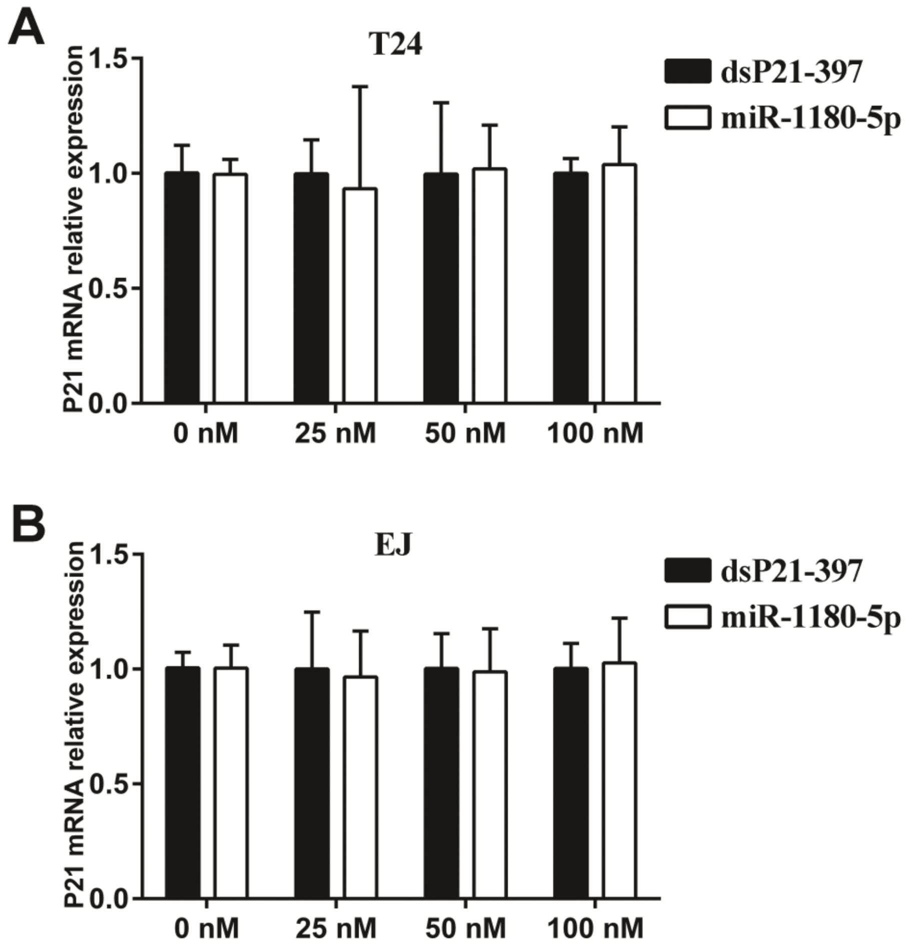

Supplement: Supplementary file 1 — Supplementary Table S1: All dsRNAs used in present study werechemically synthesized with dTdT 3'overhangs. The dsP21-397 complementary to the p21 promoter at sequences position -397 relative to the transcription starting site was used to activate p21 expression. The control dsRNA (dsControl) was designed to lack significant homology to all known human genes' sequences. The dsRNA (siP21) was used to silence p21 expression by targeting p21 mRNA 3' untranslated regions. Supplementary Table S2: The primes were used to detect p21 and GAPDH mRNA relative expression levels in BC cell lines by real-time PCR. Figure S1: In order to compare the activating activities between miR-1180-5p and dsP21-397, we transfected T24 and EJ cells with miR-1180-5p or dsP21-397 at 0nM, 25nM, 50nM and 100nM, respectively. 72 h later, p21 mRNA was determined by real-time PCR and the miR-1180-5p induced p21 mRNA expression levels were normalized to dsP21-397 groups at the same concentrations. As shown in Figure S1A and S1B, the p21 expression was not significantly different between miR-1180-5p and dsP21-397 at the same transfecting concentrations in T24 and EJ cells. [file 304753.f1.pdf]
